# Intravenous immunoglobulin‑based adjuvant therapy for severe fever with thrombocytopenia syndrome: A single‑center retrospective cohort study

**DOI:** 10.1002/jmv.70017

**Published:** 2024-11-04

**Authors:** Yu Zhai, Haopeng Li, Peng Xia, Yunfei Jiang, Hanwen Tong, Dongming Zhou, Chenxiao Jiang, Yun Liu, Jun Wang

**Affiliations:** ^1^ Department of Emergency Medicine Nanjing Drum Tower Hospital Clinical College of Xuzhou Medical University Nanjing China; ^2^ Department of Emergency Medicine Nanjing Drum Tower Hospital, Affiliated Hospital of Medical School, Nanjing University Nanjing China; ^3^ Department of Pharmacy, Nanjing Drum Tower Hospital, School of Pharmacy Nanjing Medical University Nanjing China; ^4^ Department of Hematology Nanjing Drum Tower Hospital, Affiliated Hospital of Medical School, Nanjing University Nanjing China; ^5^ Department of Pharmacy, Nanjing Drum Tower Hospital, Affiliated Hospital of Medical School Nanjing University Nanjing China

**Keywords:** intravenous immunoglobulin, mild and severe group, mortality, severe fever with thrombocytopenia syndrome

## Abstract

Intravenous immunoglobulin (IVIG) is frequently administered to patients with severe fever with thrombocytopenia syndrome (SFTS), particularly those with severe manifestations, although its efficacy remains controversial. The study retrospectively analyzed the effects of IVIG administration on SFTS patients in both mild and severe groups. The primary outcome measure was 28‐day mortality. Inverse probability of treatment weighting (IPTW) with propensity score was used to account for baseline confounders. A total of SFTS patients with complete data enrolled from January 1, 2015, to August 1, 2023. Death at 28 days occurred for 68 (17.5%) patients. By unadjusted analysis, no difference was observed for 28‐day mortality between the IVIG and non‐IVIG groups in both the mild and severe groups. Similar results were found by propensity score matching and by IPTW analysis. Although IVIG is frequently used as adjuvant therapy for severe SFTS patients, no significant association was observed between IVIG treatment and reduced mortality in this patient population.

## 1 INTRODUCTION

Severe fever with thrombocytopenia syndrome (SFTS) is an emerging tick‐borne zoonosis caused by a novel Bunyavirus. The disease is primarily prevalent in Asia, and its incidence has been increasing in recent years, with a case‐fatality rate ranging from 5% to 40% and an average mortality rate of 12.2%.^1^, ^2^ SFTS is characterized by acute fever with thrombocytopenia and leukopenia.^3^ In most cases, SFTSV infection presents as a mildly symptomatic disease, but some cases may progress to severe disease with higher mortality. The mortality rate for severe SFTS patients with multiple organ failure and central nervous system involvement is up to 44.7%,^4^ and there is a lack of effective treatment. The pathogenesis of severe SFTS primarily involves an inflammatory cytokine storm.^5^ Modulation of immune responses contributes to the recovery of multiorgan functional damage. Intravenous human immunoglobulin (IVIG) is a nonspecific immunomodulator composed of immunoglobulin G (IgG) from serum. IVIG infusion elevates plasma IgG levels and neutralizes pathogens, thereby promoting recovery. IVIG has been shown to enhance the body's immune system and mitigate pathogen‐induced damage.^6^ When endogenously produced antibodies are impaired or absent, IVIG is used to replenish antibodies or modulate the immune response in inflammatory or autoimmune diseases.^7^, ^8^ It has certain antiviral and inhibitory effects on inflammatory cytokine storms and is commonly used in critically ill patients, suggesting that IVIG shows more benefit for those patients with severe conditions, as reported in critically illCOVID‐19, severe influenza, and Middle East respiratory syndrome (MERS) patients.^9^, ^10^ Thus, physicians prefer to use IVIG in the treatment of severe SFTS patients, but there is a lack of sufficient evidence‐based support. Although the expert consensus in China suggests that IVIG can be used as an adjunctive therapy for SFTS,^11^ the heterogeneous nature of the SFTS population leads to diverse and often ineffective treatments. Therefore, identifying distinct phenotypes of SFTS patients is key to personalized management strategies. Consequently, this study retrospectively analyzed the correlation between IVIG adjuvant therapy and the prognosis of mild and severe SFTS patients to identify which subset of patients is most likely to benefit from IVIG.

## 2 MATERIALS AND METHODS

### 2.1 Study design and participants

The study protocol adhered to local regulations and received approval from the appropriate committee. The requirement for informed consent was waived.

This study retrospectively collected data on all patients with SFTS at Nanjing Drum Hospital, Jiangsu, China, from January 1, 2015, to August 1, 2023, who had confirmed SFTS. All adult patients with SFTS were screened. The exclusion criteria were as follows: (1) less than 18 years of age; (2) death within 72 h after admission; (3) a history of thyroid diseases, autoimmune diseases, malignant tumors, allergic diseases, hematological diseases, or other diseases that can cause changes in platelet count; or (4) lack of clinical data.

Based on clinical information and laboratory testing, the patients were divided into severe and mild groups.^3^, ^12^ Severe SFTS cases were defined as patients who required admission to an intensive care unit and met at least one of the following criteria: (1) acute lung injury (ALI)/acute respiratory distress syndrome (ARDS), (2) heart failure, (3) acute renal failure, (4) encephalitis, (5) shock, (6) septicemia, (7) disseminated intravascular coagulation (DIC), or (8) death.

ALI and ARDS are defined using criteria recommended by the American–European Consensus Conference on ARDS. ALI criteria: (1) Timing: Acute onset; (2) Oxygenation: PaO2/FiO2 ≤ 300 mmHg (regardless of positive end‐expiratory pressure [PEEP]); (3) Chest radiograph: Bilateral infiltrates seen on frontal chest radiograph; (4) PAWP: ≤18 mmHg when measured or no clinical evidence of left atrial hypertension. ARDS criteria (same as ALI except): Oxygenation: PaO2/FiO2 ≤ 200 mmHg (regardless of PEEP).^13^ Acute renal failure is defined as a ≥ 50% increase in serum creatinine and receipt of inpatient acute dialysis. Multiple organ dysfunction and multiple organ failure are defined using criteria as reported by Deitch.^14^ Suspected encephalitis is defined as individuals who develop a fever with at least 6 h of altered mental status, or at least 1 h of unconsciousness, seizures, or focal neurologic signs. Encephalitis is defined as the presence of decreased consciousness or seizures or altered mental status or focal neurologic signs, plus at least 2 of the following: (a) fever (>38°C); (b) abnormal cerebrospinal fluid examination (pleocytosis >5 white blood cells/mL and/or increased protein content >40 mg/dL); (c) abnormal electroencephalography findings compatible with encephalitis (e.g., diffuse or focal slow activity, or periodic lateralized epileptiform discharges); and (d) abnormal results of neuroimaging studies, including computed tomography (CT) and magnetic resonance imaging. Septic shock is defined as sepsis‐induced hypotension persisting despite adequate fluid resuscitation.^15^ DIC was scored in accordance with the International Society on Thrombosis and Haemostasis scoring system.^16^ The scoring system included platelet count (>100 × 10^9 cells/L, 0; <100 × 10^9 cells/L but >50 × 10^9 cells/L, 1; and <50 × 10^9 cells/L, 2); elevated fibrin‐related marker (no increase, 0; moderate increase, 2; and strong increase, 3) (d‐dimer was used); prolonged prothrombin time (PT) (<3 s, 0; >3 s but <6 s, 1; and >6 s, 2); and fibrinogen level (>1.0 g/L, 0; <1.0 g/L, 1). A total score of ≥5 was considered to be compatible with overt DIC.

### 2.2 Clinical data collection

The patients' information was collected, including demographic data, chronic comorbidities (including hypertension, diabetes, coronary heart disease, chronic obstructive pulmonary disease, cerebrovascular disease and immune system diseases), vital signs (cough, diarrhea, palpitations, and so on), and laboratory results (including white blood cell count, lymphocyte count, platelet count, alanine aminotransferase [ALT], aspartate aminotransferase [AST], serum creatinine, PT, d‐dimer, etc.) and the use of intravenous immunoglobulin (IVIG). The primary outcome was 28‐day mortality.

### 2.3 Statistical analysis

Statistical analysis was performed using SPSS 26.0. Values were presented as the mean (SD) or median (interquartile range [IQR]) for continuous variables as appropriate and as a percent for categorical variables. Comparisons between groups were made using the chi‐square test or Fisher's exact test for categorical variables and Student's *t*‐test or the Mann–Whitney *U* test for continuous variables, as appropriate.

### 2.4 Inverse probability of treatment weighting using the propensity score

Propensity score matching (PSM) was performed to reduce bias by adjusting for the following variables: age, sex, comorbidities, onset to admission, the use of glucocorticoids, and laboratory results on admission. PSM was implemented with a nearest‐neighbor strategy. IVIG and non‐IVIG patients were paired according to the propensity scores using exact matching with a caliper size of 0.02 and a paired ratio of 1:1.

During the matching process, a considerable proportion of patients were lost. Therefore, inverse probability of treatment weighting (IPTW) using the propensity score analysis was also performed to estimate the causal treatment effects including all eligible patients (entire cohort with complete data on all 9 covariates mentioned above).

The balance of covariates was evaluated by estimating standardized mean differences before matching, after matching, and after IPTW adjustment, with a small absolute value less than 0.25 was considered successful balancing between IVIG and non‐IVIG patients.

### 2.5 Association of IVIG therapy with 28‐day mortality

Logistic regression was performed to assess the association between IVIG therapy and 28‐day mortality. The same baseline covariates were adjusted in the Cox model. All statistical analyses were performed using R (version 4.0.0, Rstudio).

## 3 RESULTS

### 3.1 Patient characteristics

In this study, 477 patients with laboratory‐confirmed SFTSV infection were included. 88 patients were excluded owing to <3 days of hospitalization, unknown clinical outcomes, active immune diseases, unstable tumors, or other severe diseases. Consequently, 389 patients were finally included and divided into mild (*n* = 251) and severe (*n* = 138) groups. Differences in baseline and clinical characteristics are listed in Table 1.

**Table 1:** Demographic data and clinical characteristics of patients in the IVIG and non‐IVIG groups.

	Mild group (*N* = 251)			Severe group (*N* = 138)				
	IVIG (*N* = 70)	Non‐IVIG (*N* = 181)	*p* Value	IVIG (*N* = 106)	Non‐IVIG (*N* = 32)	*p* Value		
Age, years (median, IQR)	61.00 (53.00, 70.25)	58.00 (51.00, 67.00)	0.072	70.00 (57.75,75.25)	72.00 (61.50, 79.50)	0.699		
Sex (male), *n* %	26 (37.14)	93 (51.38)	0.043	53 (50.00)	18 (56.25)	0.648		
*Comorbidities, n (%)*						
Diabetes mellitus	2 (2.86)	8 (4.42)	0.438	12 (11.32)	4 (12.50)	0.855		
Hypertension	10 (14.29)	31 (17.13)	0.585	33 (31.13)	7 (21.88)	0.312		
Hepatitis	7 (10.00)	9 (4.97)	0.156	7 (6.60)	1 (3.13)	0.453		
Onset to admission, days, median (IQR)	4 (3, 6)	5 (3, 7)	0.024	4 (3.00, 6.25)	3 (2, 4)	0.139		
*Specific clinical symptoms, n (%)*							
Atypical symptoms	65 (92.86)	165 (91.16)	0.663	101 (95.28)	22 (68.75)	0.001		
Digestive symptoms	44 (62.86)	82 (45.30)	0.013	70 (66.04)	6 (18.75)	0.001		
Nervous symptoms	21 (30.00)	6 (3.31)	0.001	79 (74.53)	13 (40.63)	0.001		
Respiratory symptoms	33 (47.14)	54 (29.83)	0.01	36 (33.96)	8 (25.00)	0.340		
*Laboratory results on admission, (median, IQR)*								
WBC (10^9^/L)	2.30 (1.60, 3.50)	2.70 (1.90, 3.80)	0.69	2.20 (1.68, 3.38)	1.90 (1.20, 2.70)	0.491		
NEUT (10^9^/L)	1.40 (1.00, 2.53)	1.50 (1.00, 2.35)	0.495	1.50 (1.08, 2.20)	1.50 (0.75, 1.75)	0.388		
LYM (10^9^/L)	0.60 (0.40, 0.90)	0.80 (0.50, 1.20)	0.386	0.50 (0.40, 0.80)	0.50 (0.75, 1.75)	0.693		
PLT (10^9^/L)	60.00 (47.00, 79.25)	70.00 (50.00, 87.00)	0.123	59.00 (34.75, 78.75)	56.00 (41.00, 65.00)	0.329		
PT (s)	12.10 (11.28, 12.73)	11.50 (10.85, 12.2)	0.019	12.55 (11.80, 13.40)	11.60 (11.10, 12.80)	0.630		
APTT (s)	36.90 (33.28, 43.88)	35.40 (31.40, 39.10)	0.01	45.25 (36.73, 52.9)	38.50 (33.75, 51.05)	0.463		
D‐dimer (mg/L)	2.84 (1.78, 5.44)	1.70 (0.83, 3.48)	0.77	7.16 (2.04, 25.87)	8.30 (1.82, 14.72)	0.944		
ALT (mmol/L)	69.05 (41.15, 116.20)	55.00 (38.00, 98.85)	0.051	73.50 (42.93, 167.25)	80.50 (51.00, 143.50)	0.211		
AST (mmol/L)	159.50 (79.75, 268.55)	101.50 (62.80, 169.65)	0.001	200.75 (92.50, 608.27)	246.50 (116.70, 559.80)	0.207		
ALB (mmol/L)	27.53 (31.78, 39.95)	37.60 (34.20, 41.00)	0.176	33.15 (29.75, 40.40)	36.60 (31.50, 40.40)	0.328		
CREA (μmol/L)	65.35 (54.20, 81.78)	69.00 (56.25, 84.00)	0.824	83.95 (68.53, 129.28)	86.70 (64.05, 135.35)	0.002		
BUN (mmol/L)	5.45 (3.92, 7.50)	4.91 (3.80, 6.87)	0.21	6.17 (4.65, 9.64)	7.99 (4.39, 12.43)	0.001		
GLU(mmol/L)	6.73 (5.78, 7.84)	6.20 (5.50, 7.40)	0.549	7.10 (6.00, 8.83)	8.50 (6.15, 11.70)	0.336		
CK (U/L)	349.50 (147.75, 604.75)	225.00 (85.50, 648.00)	0.81	701.50 (314.25, 1531.50)	251.00 (130.00, 542.00)	0.581		
CKMB (U/L)	18.50 (12.75, 26.25)	16.00 (11.00, 25.00)	0.556	25.00 (11.00, 45.25)	19.00 (16.00, 29.00)	0.406		
Glucocorticoid therapy, *n* (%)	21 (30.00)	29 (16.02)	0.013	64 (60.38)	13 (40.63)	0.049		
*Outcome*								
28‐day mortality, *n* (%)	0	0		53 (50.00%)	15 (46.88%)	0.757		
The length of hospital stay,(median, IQR)	10 (7, 15)	11 (8, 15)	0.12	11 (7, 19)	11 (4, 15)	0.455		

*Note*: Atypical symptoms: Fatigue, chills, shivering, muscle aches, rash, petechiae, etc. Nervous symptoms: trembling, confusion, dysphoria, convulsion, drowsiness, coma, lethargy. Respiratory symptoms: cough, expectoration, dyspnea. Digestive symptoms: anorexia, nausea, vomit, diarrhea, abdominal pain.

Abbreviations: ALB, albumin; ALT, alanine transaminase; APTT, activated partial thromboplastin time; AST, aspartate aminotransferase; BUN, blood urea nitrogen; CK, creatine kinase; CKMB, Creatine kinase isoenzymes. CREA, creatinine; GLU, Glucose; IQR, interquartile range; IVIG, intravenous immunoglobulin; LYM, lymphocyte count; NEUT, neutrophil count; PLT, platelets; PT, prothrombin time; WBC, white blood cell.

The age of the patients in mild and severe groups treated with IVIG was 61 (53, 70.25) and 70 (57.75, 75.25), respectively. In the mild group, there were more women with IVIG treatment (26 [37.14%] vs. 93 [51.38%], *p* = 0.043). Among these patients, the time from onset to hospitalization was longer for those not receiving IVIG in the mild group (4 [3, 6] vs. 5 [3, 7], *p* = 0.024), but there was no significant difference in the severe group (4 [3, 6.25] vs. 3 [2, 4], *p* = 0.139). Neurological symptoms (such as tremors, confusion, irritability, seizures, drowsiness, and coma) were more common in the IVIG‐treated group in both the mild (21 [30%] vs. 6 [3.31%], *p* = 0.001) and severe groups (79 [74.53%] vs. 13 [40.63%], *p* = 0.001). The same conclusion was reached for digestive symptoms (such as poor appetite, nausea, vomiting, abdominal pain, and diarrhea).

Compared with patients who did not receive IVIG in the mild group, patients had higher PT (12.1 [11.28, 12.73] vs. 11.5 [10.85, 12.2], *p* = 0.019), APTT (36.9 [33.28, 43.88] vs. 35.4 [31.4, 39.1], *p* = 0.01) and AST (159.5 [79.75, 268.55] vs. 101.5 [62.8, 169.65], *p* = 0.001). In contrast, there were no statistically significant differences between the mild group patients treated with or without IVIG regarding age, comorbidities, concurrent diabetes mellitus, hypertension, hepatitis, WBC count, neutrophil count, lymphocyte count, platelet count, ALT level, serum creatinine level, BUN level, GLU level, ALB level, d‐dimer level, CK, and CK‐MB levels. In the severe group, patients treated with or without IVIG showed no statistically significant differences in age, gender, concurrent hypertension, diabetes mellitus, hepatitis, white blood cell count, neutrophil count, lymphocyte count, platelet count, PT, APTT, d‐dimer, ALT, AST, ALB, GLU, CK, and CK‐MB levels. In addition, patients who received IVIG therapy in the severe group had a lower CR (83.95 [68.53, 129.28] vs. 86.7 [64.05, 135.35], *p* = 0.002) and BUN (6.17 [4.65, 9.64] vs. 7.99 [4.39, 12.43], *p* = 0.001). More patients in the mild group used glucocorticoids (21 [30%] vs. 29 [16.02%], *p* = 0.013) without IVIG, but conversely, more patients in the severe group used glucocorticoids with IVIG (64 [60.38%] vs. 13 [40.63%], *p* = 0.049). Glucocorticoid therapy was administered with significantly higher frequencies with IVIG in both groups.

**Figure 1:**
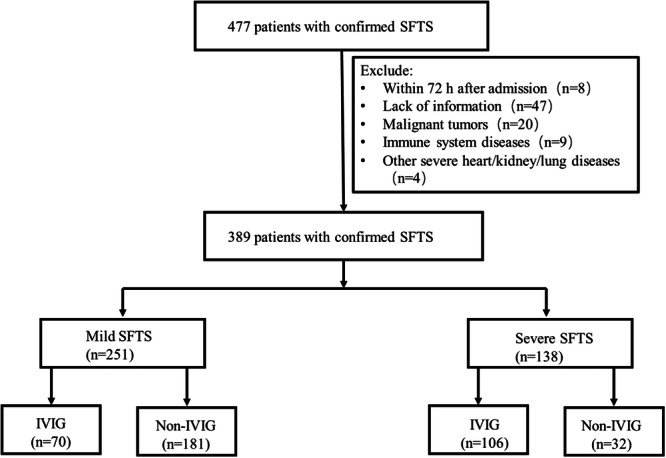
Flowchart of the research. IVIG, intravenous immunoglobulin; SFTS, severe fever with thrombocytopenia syndrome.

To further explore the relationship between death outcomes and IVIG in the severe group, the data of fatal and nonfatal cases with IVIG in the severe group were analyzed (Table 2). In all patients with IVIG, the fatal group exhibited more gastrointestinal and nervous symptoms. At the same time, they had more complications such as shock, encephalitis, ALI/ARDS, and heart failure. A significant difference in the length of hospital stay was observed between the fatal and nonfatal groups with IVIG (*p* < 0.002) (Figure 1).

**Table 2:** Characteristics of patients with Nonfatal and fatal cases in the severe cases admitted with SFTSV infection.

Severe group			
	Nonfatal (*n* = 54)	Fatal (*n* = 52)	*p* Value
Age, years, Mean ± SD	64.426 ± 10.812	68.135 ± 10.544	0.077
Sex (Male), *n* %	27 (50%)	26 (50%)	1.000
Comorbidities, *n* (%)			
Diabetes mellitus	4 (7.41%)	8 (15.38%)	0.200
Hypertension	16 (29.63%)	17 (32.69%)	0.730
Hepatitis	6 (11.11%)	3 (5.77%)	0.320
Onset to admission, days, median (IQR)	4.00 (3.00,6.00)	4.00 (3.00, 5.25)	0.845
Specific clinical symptoms, *n* (%)			
Atypical symptoms	52 (96.30%)	48 (92.31%)	0.370
Digestive symptoms	34 (62.96%)	49 (94.23%)	<0.001
Nervous symptoms	33 (61.11%)	46 (88.46%)	0.001
Respiratory symptoms	25 (46.30%)	22 (42.31%)	0.160
*Complications, n (%)*			
Shock	6 (11.11%)	23 (44.23%)	<0.001
Encephalitis	12 (22.22%)	22 (42.31%)	0.027
ALI/ARDS	7 (12.96%)	35 (69.23%)	<0.001
Renal failure	3 (5.55%)	9 (17.31%)	0.056
Heart failure	1 (1.85%)	7 (13.46%)	0.026
Septicemia	5 (9.26%)	11 (21.15%)	0.087
DIC score, Mean ± SD	5.03 ± 0.17	5.08 ± 0.27	0.471
The dose of IVIG, g, median (IQR)	65.0 (30.0, 100.0)	50 (27.5, 82.5)	0.111
The course of IVIG, g, median (IQR)	5 (4−6)	5 (4−6)	0.237
Onset to the use of IVIG, median (IQR)	1 (1, 2)	1 (1, 2)	0.617
Glucocorticoid therapy, n (%)	31 (57.41%)	33 (63.46%)	0.520
HSA infusion, *n* (%)	36 (66.67%)	41 (78.85%)	0.160
G‐CSF, *n* (%)	37 (68.52%)	31 (59.62%)	0.340
Plasma infusion, *n* (%)	34 (62.96%)	39 (75.00%)	0.180
*Laboratory results on admission, median (IQR) or mean ± SD*			
WBC (10^9^/L)	2.30 (1.400, 4.072)	2.15 (1.800, 2.950)	0.344
NEUT (10^9^/L)	1.4 (0.800, 2.600)	1.5 (1.175, 2.200)	0.324
LYM (10^9^/L)	0.5 (0.400, 0.800)	0.5 (0.375, 0.800)	0.344
PLT (10^9^/L)	54.000 ± 29.858	60.173 ± 30.853	0.298
PT (s)	12.383 ± 1.170	12.663 ± 1.314	0.249
APTT (s)	44.20 (39.175, 48.950)	44.85 (38.275, 51.175)	0.160
D‐dimer (mg/L)	6.925 (2.808, 14.967)	8.890 (2.232, 20.820)	0.334
ALT (mmol/L)	79 (41.625, 131.200)	75 (48.525, 125.850)	0.727
AST (mmol/L)	185.00 (100.250, 378.075)	194.55 (127.55, 576.25)	0.319
ALB (mmol/L)	34.540 ± 5.632	33.615 ± 5.005	0.375
CREA (μmol/L)	76.2 (65.625, 98.800)	85.9 (73.000, 124.325)	0.038
BUN (mmol/L)	5.905 (4.500, 7.947)	6.31 (5.00, 9.24)	0.145
GLU (mmol/L)	6.6 (5.655, 8.982)	7.3 (6.030, 9.100)	0.146
CK (U/L)	612.5 (219.50, 1131.75)	477 (225.75, 1073.25)	0.893
CKMB (U/L)	22.5 (14.25, 36.25)	22.5 (12.75, 39.50)	0.629
*Outcome*			
The length of hospital stay,(median, IQR)	18 (13.5, 22.5)	7.5 (4.0, 10.0)	0.002

*Note*: Data presented as *n* (%) or median. IQR unless otherwise noted. Atypical symptoms: Fatigue, chills, shivering, muscle aches, rash, petechiae, etc. Nervous symptoms: trembling, confusion, dysphoria, convulsion, drowsiness, coma, lethargy. Respiratory symptoms: cough, expectoration, dyspnea. Digestive symptoms: anorexia, nausea, vomit, diarrhea, abdominal pain.

Abbreviations: ALB, albumin; ALT, alanine transaminase; APTT, activated partial thromboplastin time; AST, aspartate aminotransferase; BUN, blood urea nitrogen; CK, creatine kinase; CKMB, Creatine kinase isoenzymes; CREA, creatinine; G‐CSF, granulocyte colony‐stimulating factor; GLU: Glucose; HSA, human serum albumin; IVIG, intravenous immunoglobulin; LYM, lymphocyte count; NEUT, neutrophil count; PLT, platelets; PT, prothrombin time; WBC, white blood cell.

### 3.2 The outcome

**Figure 2:**
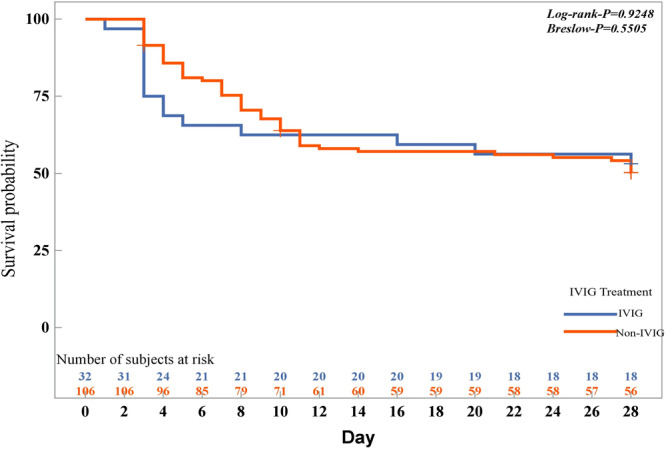
Kaplan–Meier analysis for 28‐day survival of the IVIG and non‐IVIG groups in the severe group. IVIG, intravenous immunoglobulin.

All patients in the mild group were survived. The 28‐day mortality of the severe group was 49.28%. There were no significant differences with IVIG in the number of deaths by 28 days (Table 1). Kaplan–Meier curves for estimated survival did not show any significant differences in the outcome of the severe group (Figure 2).

### 3.3 Propensity score‐matched analysis and IPTW using the propensity score

The PSM resulted in 24 patients who received IVIG matched to 24 patients who did not receive IVIG. More participants received IVIG than those who did not; thus, 82 IVIG patients were unmatched in contrast to 8 non‐IVIG patients in the severe group. In the propensity score‐matched analysis, IVIG therapy was not associated with differences in 28‐day mortality, nor in a logistic regression model (Table 3).

**Table 3:** 28‐day mortality of patients confirmed SFTS using various adjustment methodologies.

Cohorts	Logistic regression model^a^		Cox proportional hazards regression model^b^	
	aOR (95% CI)	*p* Value	aHR (95% CI)	*p* Value
Original cohort	1.09 (0.49, 2.41)	0.829	0.97 (0.55, 1.73)	0.926
PSM cohort	1.67 (0.53, 5.27)	0.384	1.52 (0.64, 3.61)	0.347
IPTW cohort	1.71 (1.04, 2.85)	0.053	1.41 (0.96, 2.05)	0.077

Abbreviations: aHR, adjusted hazard ratio; aOR, adjusted odds ratio; IPTW, inverse probability of treatment weighting; PSM, propensity score matching.

^a^ The logistic regression model was adjusted for the use of glucocorticoids, age, sex, and history of hypertension, diabetes, hepatitis, WBC, NEUT, LYM, PLT, D‐dimer, PT, APTT, ALT, AST, BUN, CK, CK‐MB;

^b^ The Cox proportional hazards regression model was adjusted for the same abovementioned baseline covariates.

IPTW, assessed from patients with complete covariate data included in the propensity analysis, also resulted in between‐group balance on baseline characteristics. The logistic regression model did not show a significant difference between IVIG treatment and 28‐day mortality compared with the control non‐IVIG group (Table 3).

## 4 DISCUSSION

This study investigated the correlation between IVIG application and the prognosis of mild and severe SFTS patients. The results showed that 28% of mild and 77% of severe patients received IVIG therapy, illustrating the fact that in clinical practice, clinicians prefer to use IVIG treatment in severe SFTS patients. However, multivariate logistic analysis showed that the use of IVIG was not correlated with poor prognosis in patients with mild or severe SFTS. The IVIG group presented with more specific symptoms like digestive and nervous symptoms which are independent risk factors for SFTS.^17^, ^18^ Additionally, the IVIG group received more glucocorticoid treatment, suggesting that the condition of patients in the IVIG group was more severe.

Treatment with IVIG has the potential effects of anti‐inflammation and passive immunity, which provide a rationale for the use of IVIG in severe infections. IVIG has been used for the treatment of bacterial infection, sepsis, and severe viral infection. Its efficacy has been confirmed in viral infections, but since IVIG is not a specific antibody for any virus, some researchers hold a doubtful view of its usage in acute virus infections.^19^, ^20^, ^21^, ^22^ IVIG affects lymphocyte differentiation and maturation, blocks normal leukocyte immune responses, suppresses inflammatory injury, and inhibits cytokine production.^23^, ^24^, ^25^ It has been shown to improve the body's defense system, block related receptors in target cells, and prevent pathogens from further damaging target cells.^6^ Based on the clinical efficacy of IVIG in the treatment of severe influenza^23^, ^26^ and coronaviruses such as SARS and MERS,^27^ it has been postulated that IVIG is beneficial for SFTS patients as well. Previous studies indicate that severe SFTS patients had higher SFTS viral loads in serum than mild SFTS patients.^18^ Both IL‐6 and IL‐10 significantly increase in SFTS patients.^12^, ^28^ SFTSV infection induces a cytokine storm with abnormally expressed cytokine profiles, which might be associated with disease severity.^29^, ^30^, ^31^ The concentration of IL‐6 in plasma is dramatically decreased in COVID‐19 patients treated with IVIG, suggesting that the benefits of IVIG are associated with reduced inflammation.^9^ Thus, from a mechanistic perspective, IVIG may benefit severe SFTS patients. There is no consensus on the efficacy of IVIG treatment in SFTS, and related research is rather limited. Kim et al. and Denic et al. reported cases of severe SFTS patients with complications in which combined treatment with IVIG and other drugs showed positive therapeutic effects.^32^, ^33^, ^34^ Liu conducted research confirming that the fatality rate in SFTS patients with neurological complications decreased when IVIG was over 80 g longer than 5 days.^35^ Zhang et al. conducted a multicenter, retrospective cohort study of IVIG treatment of SFTS patients; while no obvious efficacy of IVIG in saving lives or improving outcomes of SFTS was observed. However, a weakness of the study was it did not consider the heterogeneity of the severity of illness among participants.^36^ The study concluded an adverse effect of IVIG treatment since the mortality rate in the IVIG group was higher than in the non‐IVIG group, which can be explained by the fact that physicians prefer to use IVIG in severe patients. Thus, in this study, all patients were divided into mild and severe groups to assess whether the effectiveness differed in these two phenotypic subgroups. Still, the multivariate logistic analysis in our study manifested no correlation.

Given the absence of fatalities among patients with mild SFTS, the use of IVIG for treating mild cases is not recommended due to its high cost. In contrast, glucocorticoid treatment may be beneficial for patients with mild SFTS and is a more cost‐effective option. The use of IVIG in severe patients still needs further investigation since the cytokine storm is a major cause of severe SFTS, and the mechanism underlying the lack of effectiveness of IVIG in severe patients can be explained from three perspectives: First, the IVIG used in clinical practice may not contain specific antibodies against SFTSV, thus failing to provide protection against SFTSV infection. Even in patients from highly endemic regions with previous SFTSV infection, the antibody response to SFTSV is elicited at low levels and is not long‐lasting. Secondly, since the host immune response plays a crucial role in determining the severity and clinical outcomes of SFTSV infection^30^, ^37^ and IVIG can target the immune response, severe SFTS patients may benefit from IVIG treatment.^9^ However, in severe SFTS patients, with excessive inflammatory responses, the efficacy of IVIG treatment will be significantly limited.^9^, ^38^, ^39^ Thirdly, severe SFTS involves multiple organs, including the lungs, heart, kidneys, brain, and hematological system which necessitates exploring various treatment approaches. IVIG is only one of the attempts so it is difficult to judge the therapeutic effect of IVIG on the whole cohort. The only proven effect of IVIG in SFTS may be limited to patients with encephalitis and neurological symptoms.^32^, ^35^ We further conducted a subgroup analysis of fatal and nonfatal groups in severe SFTS patients, manifesting that in the fatal group, patients presented with more digestive and nervous symptoms, which is consistent with previous studies.^17^, ^18^ Among the different severity grouping criteria, patients classified into the severe group due to shock, ALI/ARDS, and encephalitis are more likely to have fatal outcomes, which is consistent with previous study.^40^, ^41^, ^42^, ^43^ In addition, we found that patients who developed heart failure were more likely to have fatal outcomes. The occurrence of heart failure may be caused by myocarditis, identified in previous studies as an early risk factor for SFTS patients. In the future, we need to expand the sample size to further verify this conclusion.^44^

Therefore, for those patients complicated with shock, ALI/ARDS, encephalitis, and heart failure, more effective treatments should be explored beyond IVIG, and physicians should pay more attention. The timing and dosing of IVIG treatment in the severe group were also analyzed, showing no statistical difference between the fatal and nonfatal groups, although the dosing of IVIG was relatively higher in the nonfatal group. Further prospective analysis is needed to confirm the proper dosing for treating severe patients.

This study had some limitations. First, it was a single‐center retrospective cohort study of 389 patients, lacking sufficient representativeness. Second, as an observational study, unmeasured confounders and residual measured confounders may have influenced the results despite effective PSM and IPTW. Third, the evaluation of the immunoglobulin effect is mainly based on clinical manifestations, rather than direct cellular and molecular assessment, including viral load and lymphocyte activation. With the progression in understanding SFTS, rigorously designed prospective studies and more developed evaluation systems are needed to confirm the efficacy of IVIG in SFTS treatment.

## 5 CONCLUSION

The present study is the first clinical research evaluating the efficacy of IVIG in mild and severe SFTS patients. The data showed IVIG is not associated with the mortality rate in mild or severe SFTS patients. These relationships need to be confirmed by prospective randomized controlled studies.

## AUTHOR CONTRIBUTIONS

Jun Wang, Yun Liu, and Chenxiao Jiang conceived of the research and designed the study. Dongming Zhou acquired the funding. Yu Zhai, Haopeng Li, and Yun Liu wrote the manuscript. Yu Zhai, Peng Xia, Yunfei Jiang, Hanwen Tong and Haopeng Li organized the database. Yu Zhai and Dongming Zhou analyzed the data. All authors have read and agreed to the published version of the manuscript.

## CONFLICT OF INTEREST STATEMENT

The authors declare no conflict of interest.

## Supporting information

Supporting information.

## Data Availability

The data that support the findings of this study are available on request from the corresponding author. The data are not publicly available due to privacy or ethical restrictions.
