# Supplementary material for: Intravenous immunoglobulin‑based adjuvant therapy for severe fever with thrombocytopenia syndrome: A single‑center retrospective cohort study
Source: J Med Virol. 2024 Nov 4;96(11):e70017. doi: 10.1002/jmv.70017 (PMC11600480; doi:10.1002/jmv.70017)
Supplement: Supplementary file 1 — Supporting information. [file JMV-96-e70017-s001.docx]

**Supplementary table 1** Clinical characteristics of the IVIG group and non-IVIG group after propensity score matching adjustment.

| **Severe Group(N=48)** | | | | |
| --- | --- | --- | --- | --- |
|  | **IVIG（N=24）** | **Non-IVIG（N=24）** | **P** | **SMD** |
| **Age, years (median, IQR)** | 70.0(61.5,76.0) | 66.5(53.5,76.0) | 0.475 | 0.258 |
| **Sex (Male), n %** | 13(54.17) | 11(45.83) | 0.564 | 0.166 |
| **Comorbidities, n (%)** |  |  |  |  |
| Diabetes mellitus | 7(29.17) | 5(20.83) | 0.505 | 0.189 |
| Hypertension | 4(16.67) | 4(16.67) | 1.000 | 0 |
| Hepatitis | 3(12.50) | 1(4.17) | 0.602 | 0.243 |
| **Onset to admission, days, median (IQR)** | 4.29±1.76 | 4.13±1.85 | 0.750 | 0.074 |
| **Specific clinical symptoms, n (%)** | | | | |
| Atypical symptoms | 23(95.83) | 21(87.50) | 0.602 | 0.250 |
| Gastrointestinal symptoms | 11(45.83) | 14(58.33) | 0.386 | 0.250 |
| Nervous symptoms | 13(54.17) | 9(37.50) | 0.247 | 0.230 |
| Respiratory symptoms | 5(20.83) | 7(29.17) | 0.505 | 0.170 |
| **Laboratory results on admission, (median, IQR)** | | | | |
| WBC (10^9^/L) | 2.2 (1.3,2.7) | 1.7 (1.2,2.4) | 0.604 | 0.151 |
| NEUT (10^9^/L) | 1.5 (0.8,2.1) | 1.0 (0.8,1.7) | 0.737 | 0.097 |
| LYM (10^9^/L) | 0.4 (0.3,0.6) | 0.5 (0.3,0.7) | 0.462 | 0.214 |
| PLT (10^9^/L) | 52.5(30.5,61.75) | 46.0(32.0,65.0) | 0.579 | 0.141 |
| PT (s) | 12.6 (11.8,13) | 12.25 (11.3,12.9) | 0.961 | 0.014 |
| APTT (s) | 47.85 (38.2,54.7) | 38.05 (36.3,45.6) | 0.663 | 0.127 |
| D-dimer (mg/L) | 9.72 (2.98,20.49) | 7.57 (1.09,16.98) | 0.810 | 0.070 |
| ALT (mmol/L) | 85.25 (59,175) | 62.25 (41,170) | 0.778 | 0.082 |
| AST (mmol/L) | 207.5 (105,631) | 181.25 (81,424) | 0.890 | 0.040 |
| ALB (mmol/L) | 35.25 (30.5,39.0) | 36.85 (31.7,39.1) | 0.892 | 0.039 |
| CREA (μmol/L) | 98.9 (67.9,124.0) | 79.35 (52.9,98.8) | 0.767 | 0.086 |
| BUN (mmol/L) | 7.92 (5.12-9.10) | 6.66 (5.00,8.74) | 0.964 | 0.013 |
| GLU (mmol/L) | 6.75 (5.53,7.90) | 7.25 (6.10,8.50) | 0.826 | 0.064 |
| CK (U/L) | 544.5(208.0,1136.0) | 492.0(187.5,1232.0) | 0.818 | 0.273 |
| CKMB (U/L) | 24.5 (16,29) | 15.5 (11,35) | 0.409 | 0.241 |
| **Glucocorticoid therapy,n (%)** | 14(58.33) | 12(50.00) | 0.562 | 0.169 |
| **Outcome** |  |  |  |  |
| 28-day mortality, n (%) | 12(50.00) | 9(37.50) | 0.561 | 0.254 |

Corrected by age; sex; comorbidities; onset to admission; the use of glucocorticoids; laboratory results on admission.

SMD, standardized mean differences; IQR, interquartile range; IVIG, intravenous immunoglobulin; NEUT, neutrophil count; WBC, white blood cell; LYM, lymphocyte count; PLT, platelets; PT, prothrombin time; APTT, activated partial thromboplastin time; ALT, alanine transaminase; AST, aspartate aminotransferase; ALB, albumin; CREA, creatinine; BUN, blood urea nitrogen; GLU: Glucose; CK, creatine kinase; CKMB, Creatine kinase isoenzymes.

Atypical symptoms: Fatigue; chills; shivering; muscle aches; rash; petechiae, etc.

Nervous symptoms: trembling; confusion; dysphoria; convulsion; drowsiness; coma; lethargy.

Respiratory symptoms: cough; expectoration; dyspnea.

Digestive symptoms: anorexia; nausea; vomit; diarrhea; abdominal pain.

| **Severe Group(N=138)** | | | | |
| --- | --- | --- | --- | --- |
|  | **IVIG（N=106）** | **Non-IVIG（N=32）** | **P** | **SMD** |
| **Age, years (median, IQR)** | 68.5(38,87) | 69.5(49,83) | 0.549 | 0.120 |
| **Sex (Male), n %** | 53(50) | 17(53.12) | 0.914 | 0.063 |
| **Comorbidities, n (%)** |  |  |  |  |
| Diabetes mellitus | 33(31.13) | 7(21.88) | 0.430 | 0.211 |
| Hypertension | 12(11.32) | 4(12.5) | 1.000 | 0.036 |
| Hepatitis | 9(8.49) | 1(3.12) | 0.524 | 0.231 |
| **Onset to admission, days, median (IQR)** | 4(3,6) | 4(3,5.75) | 0.249 | 0.248 |
| **Specific clinical symptoms, n (%)** | | | | |
| Atypical symptoms | 101(95.28) | 25(78.12) | 0.008 | 0.522 |
| Gastrointestinal symptoms | 70(66.04) | 16(50) | 0.152 | 0.329 |
| Nervous symptoms | 79(74.53) | 12(37.5) | 0.001 | 0.804 |
| Respiratory symptoms | 47(44.34) | 10(31.25) | 0.266 | 0.272 |
| **Laboratory results on admission, (median, IQR)** | | | | |
| WBC (10^9^/L) | 2.25 (1.5,3.6) | 2.2 (1.4,3.6) | 0.341 | 0.161 |
| NEUT (10^9^/L) | 1.5 (1.0,2.4) | 1.45 (0.8,2.3) | 0.288 | 0.175 |
| LYM (10^9^/L) | 0.5 (0.4,0.8) | 0.5 (0.3,0.9) | 0.565 | 0.113 |
| PLT (10^9^/L) | 55(34.50,71.75) | 47(37.00,65.00) | 0.482 | 0.147 |
| PT (s) | 12.5 (11.7,13.3) | 12.05 (11.2,13.1) | 0.812 | 0.041 |
| APTT (s) | 44.3 (38.5,49.7) | 39.45 (36.3,52.3) | 0.756 | 0.056 |
| D-dimer (mg/L) | 8.38 (2.30,17.77) | 7.09 (1.09,11.74) | 0.893 | 0.026 |
| ALT (mmol/L) | 78 (46,130) | 68.75 (38,199) | 0.425 | 0.153 |
| AST (mmol/L) | 185.55 (105,437) | 224.55 (81,533) | 0.416 | 0.159 |
| ALB(mmol/L) | 33.55 (30.2,37.4) | 36.85 (30.1,39.0) | 0.344 | 0.194 |
| CREA (μmol/L) | 81.1 (67.4,110.0) | 92.4 (63.1,147.3) | 0.007 | 0.417 |
| BUN (mmol/L) | 6.14 (4.59,8.42) | 7.28 (5.69,12.06) | 0.003 | 0.501 |
| GLU(mmol/L) | 7.15 (5.80,9.01) | 6.7 (5.90,8.40) | 0.330 | 0.217 |
| CK (U/L) | 544.5(216.25,1131.75) | 492(189.75,1082.50) | 0.640 | 0.094 |
| CKMB (U/L) | 22.5 (13,38) | 16 (12,36) | 0.506 | 0.124 |
| **Glucocorticoid therapy,n (%)** | 64(60.38) | 14(43.75) | 0.144 | 0.338 |
| **Outcome** | | | | |
| 28-day mortality, n (%) | 52(49.06) | 15(46.88) | 0.988 | 0.044 |

**Supplementary table 2** Clinical characteristics of the IVIG group and non-IVIG group adjusted by inverse probability of treatment weighting.

Corrected by age; sex; comorbidities; onset to admission; the use of glucocorticoids; laboratory results on admission.

SMD, standardized mean differences; IQR, interquartile range; IVIG, intravenous immunoglobulin; NEUT, neutrophil count; WBC, white blood cell; LYM, lymphocyte count; PLT, platelets; PT, prothrombin time; APTT, activated partial thromboplastin time; ALT, alanine transaminase; AST, aspartate aminotransferase; ALB, albumin; CREA, creatinine; BUN, blood urea nitrogen; GLU: Glucose; CK, creatine kinase; CKMB, Creatine kinase isoenzymes.

Atypical symptoms: Fatigue; chills; shivering; muscle aches; rash; petechiae, etc.

Nervous symptoms: trembling; confusion; dysphoria; convulsion; drowsiness; coma; lethargy.

Respiratory symptoms: cough; expectoration; dyspnea.

Digestive symptoms: anorexia; nausea; vomit; diarrhea; abdominal pain.
